# Supplementary material for: Effect of Type 2 Diabetes and Impaired Glucose Tolerance on Digestive Enzymes and Glucose Absorption in the Small Intestine of Young Rats
Source: Nutrients. 2022 Jan 17;14(2):385. doi: 10.3390/nu14020385 (PMC8779211; doi:10.3390/nu14020385)
Supplement: Supplementary file 1 [file nutrients-14-00385-s001.zip › nutrients-1512336-supplementary.pdf]

Table S1. List of reagents

Type of analysis	Reagents
**Biochemical determination of enzymatic activities: glucoamylase, maltase**	T1503 Trizma base, Sigma; P 8250 Peroxidase, type ll, activity 150-200 u/mg, Sigma; M 5885 Maltose, Sigma; G 6125 Glucose oxidase, type ll, activity 15-20 ku/g, Sigma; G 8270 Glucose, min 99.5%, Sigma.
**alkaline phosphatase**	71768 4-Nitrophenyl phosphate disodium salt hexahydrate, Fluka.
**aminopeptidase N**	A 2628 L-alanine -naphthylamide, free base, Sigma.
**Immunohistochemical determination of glucose transporters in the enterocytes**	SC 9117 GLUT2 (H-67) rabbit polyclonal Jg G, Santa Cruz Biotechnology; SC 98974 SGLT1 (H- 85) rabbit polyclonal Jg G, Santa Cruz Biotechnology; ab 97064 DnkpAb to Rb Jg G (HRP), Abcam; PA1- 74362 Goat anti-rabbit Jg G secondary antibody (HRP conjugate), Thermo scientific Pierce Protein Biology Products.
**Assessment of glucose absorption in the small intestine**	G8270 D-Glucose, Sigma-Aldrich.
**Induction of type 2 diabetes**	S0130 Streptozotocin, Sigma-Aldrich LLC., St. Louis, MO, USA Sigma.
**OGTT and ITT tests**	G8270 D-Glucose, Sigma-Aldrich; Insulin HUMALOG, Lilly France, USA.
